# Spotlight on a New Heme Oxygenase Pathway: Testosterone-Induced Shifts in Cardiac Oxidant/Antioxidant Status

**DOI:** 10.3390/antiox8080288

**Published:** 2019-08-07

**Authors:** Renáta Szabó, Denise Börzsei, Krisztina Kupai, Alexandra Hoffmann, Rudolf Gesztelyi, Anikó Magyariné Berkó, Csaba Varga, Anikó Pósa

**Affiliations:** 1 Department of Physiology, Anatomy and Neuroscience, Faculty of Science and Informatics, University of Szeged, 6726 Szeged, Hungary; 2 Department of Physiology, Anatomy and Neuroscience, Interdisciplinary Excellence Centre, University of Szeged, 6726 Szeged, Hungary; 3 Department of Pharmacology and Pharmacotherapy, University of Debrecen, 4032 Debrecen, Hungary

**Keywords:** heme oxygenase, testosterone, inflammation, antioxidant

## Abstract

A low testosterone level contributes to the development of oxidative damages; however, the cardiovascular effects of exogenous hormone therapy are not well elucidated. The aim of our work is to study the association of the testosterone level, antioxidant/oxidant system, and anti-inflammatory status related to the heme oxygenase (HO) system. To determine the effects of testosterone, 10-week-old, and 24-month-old sham-operated and castrated male Wistar rats were used. One part of the castrated animals was daily treated with 2.5 mg/kg cyproterone acetate, while the hormone replacement therapy was performed via an *i.m.* injection of a dose of 8.0 mg testosterone undecanoate/kg/once a week. The plasma testosterone level, the activity of HO and myeloperoxidase (MPO) enzymes; the concentrations of the HO-1, tumor necrosis alpha (TNF-α), and cyclic guanosine monophosphate (cGMP), as well as the total level of glutathione (GSH + GSSG) were determined from the cardiac left ventricle. In accordance with the testosterone values, the aging process and castration resulted in a decrease in antioxidant HO activity, HO-1 and cGMP concentrations and in the level of GSH + GSSG, whereas the inflammatory TNF-α and MPO activity significantly increased. Testosterone therapy was able to restore the physiological values. Our results clearly show that testosterone replacement therapy increases the antioxidant status and mitigates the inflammatory parameters via the modulation of the HO system.

## 1. Introduction

Low testosterone level and testosterone deficiency have been associated with the incidence and progression of chronic diseases, such as metabolic syndrome [1] and cardiovascular diseases (CVDs) [2], which are the most the common cause of death worldwide. Testosterone may contribute to the reduction of myocardial infarction and protects the heart against ischemic injury. It has also been shown that testosterone [3], as well as estrogen, can modulate nitric oxide release, and consequently influence the vasodilatory mechanisms on blood vessels [4]. While the cardioprotective and vasodilatory actions have been well documented, the hormonal effects on inflammatory processes and oxidant/antioxidant homeostasis have not been fully elucidated, and the studied results are controversial. Chignalia et al. reported that testosterone induces reactive oxygen species (ROS) generation and vascular smooth muscle cell migration by NADPH oxidase under hypertensive conditions [5]. In addition to the effects of NADPH oxidase, androgens also modulate the mitochondrial ROS generation [6]. Beside the prooxidant effects, numerous studies verify the antioxidant capacity of testosterone in the cardiovascular system. Zhang et al. demonstrated that testosterone replacement restored the antioxidant capacity of the heart [7]. As a result of the discrepant outcomes, further investigations of various possible contributing factors are necessary to study the role of testosterone on oxidant/antioxidant mechanisms.

According to our best knowledge, no reports have been published concerning the relationship of the heme oxygenase (HO) enzyme system and testosterone level in the myocardium; however, its cardioprotective effects are significant. HO is a key rate-limiting enzyme in the regulation of cellular oxidative stress. It catalyzes the degradation of heme and generates biliverdin/bilirubin, free iron and carbon monoxide (CO), which play a key role in the defense and repair of oxidative stress-induced damage [8]. The induction of HO-1 is considered to enhance the antioxidant capacity of cells to provide protection against oxidative stress [9]. While biliverdin and bilirubin are efficient in the scavenging of ROS, CO contributes significantly to the reduction of pro-inflammatory cytokines [10]. Based on the antioxidant and anti-inflammatory properties of HO, an examination of the enzyme system might be a candidate for the understanding of the oxidative status in different testosterone-saturated conditions.

Oxidative stress is involved in age-related dysfunctions and the decline in the testosterone hormone level and may induce progressive oxidative damages in the cardiovascular system. The aim of our work is to study the association between the testosterone level and oxidative/inflammatory processes in male rats subjected to testosterone deprivation (young castrated and aged rats) and testosterone replacement. To add a significant complexity to the interaction between the testosterone level and oxidative mechanisms, the HO enzyme activity and concentration as well as the inflammatory parameters are analysed in the heart.

## 2. Materials and Methods

### 2.1. Experimental Protocol

In our study, 10-week-old (young), and 24-month-old (aged) male Wistar rats were used and housed (Directive 2010/63/EU) at a controlled temperature (20–23 °C) on a 12 h light-dark cycle. Food and water were available *ad libitum.* All procedures were approved by the Institutional Ethical Committee and were performed in accordance with the standards of the European Community guidelines on the care and use of laboratory animals.

**Figure 1:**
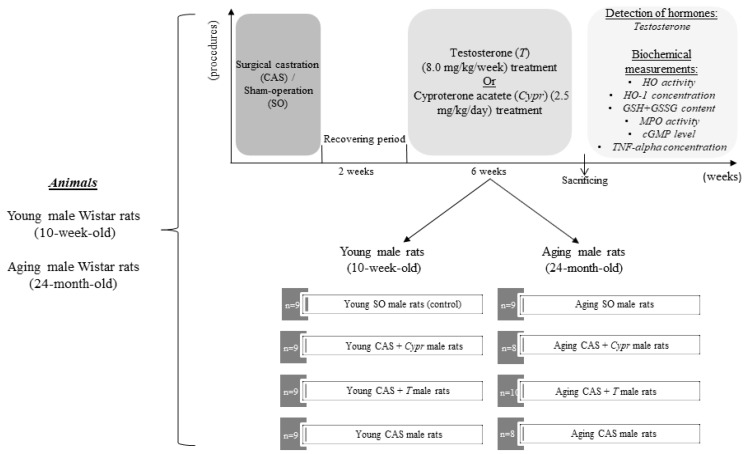
The experimental protocol of the study. CAS = surgical castration, SO = sham operation, T = testosterone replacement therapy, Cypr = cyproterone acetate treatment, HO = heme oxygenase enzyme, HO-1 = heme oxygenase-1, GSH + GSSG = reduced glutathione/oxidized glutathione, MPO = myeloperoxidase enzyme, and cGMP = cyclic guanosine monophosphate.

Both young and aged rats were randomly divided into the following groups: castrated or control (sham-operated) animals. During castration surgery, the spermatic cord was tied, the testes were removed, and the incision was provided by penicillin. The incision was closed, and the rats were allowed to recover. After a 2-week-recovery period, one part of the young and aged castrated animals was administrated cyproterone acetate (Bayer AG, Berlin, Germany; 2.5 mg/kg per day, *oral treatment*) in order to block the androgen production of the adrenal glands, while the other part of the castrated rats was treated with testosterone to restore the physiological hormone levels [11]. The testosterone replacement was performed for 6 weeks via an intramuscular injection of a dose of 8.0 mg/kg testosterone undecanoate (Nebido, Bayer Schering Pharma AG, Berlin, Germany) once a week dissolved in soybean oil. The groups that did not receive testosterone replacement were administrated the same volume of steroid vehicle. The number of the animals per group is detailed in Table 1. Following the 6-week-experimental period, the rats in each group were sacrificed. The serum testosterone and blood parameters were immediately measured, while the heart left ventricle (LV) samples were excised, frozen, and kept at −80 °C until the biochemical analyses. In our experiment, all efforts were made to minimize the number of animals as well as the animal’s suffering. The experimental protocol of the study is shown in Figure 1.

### 2.2. Measurement of Serum Testosterone, GOT, GPT, Cholesterol and Triglyceride Levels

The serum levels of total testosterone were analyzed by Immulite 2000XPi (Siemens) chemiluminescent immunoassay, whereas the glutamic oxaloacetic transaminase (GOT), glutamic-pyruvic transaminase GPT), cholesterol, and triglyceride levels were measured on the Biolis 24i Premium system (Siemens) at the end of the 6-week-experimental period. The reactions of GOT and GPT were monitored by measuring the rate of decrease in absorbance at 340 nm due to the oxidation of NADH into NAD and were expressed as U/L. The triglyceride and total cholesterol levels were measured at 546 nm and expressed as mmol/L.

### 2.3. Measurement of HO Activity

The cardiac LV samples were homogenized in an ice cold buffer [10.0 mM N–2–hydroxyethylpiperazine–N′–2-ethanesulfonic acid, 0.10 mM ethylenediaminetetraacetic acid disodium salt dihydrate, 1.0 mM dithiotreitol (DTT), 32.0 mM sucrose, 10.0 μg/mL trypsin inhibitor, 2.0 μg/mL aprotinin, 10.0 μg/mL leupeptin; pH7.4], centrifuged at 15,000× *g* for 20 min at 4 °C, and the remaining supernatant was used for the measurements. The reaction mix consisted of a 75 µL sample, 2.0 mM glucose–6–phosphate, 0.14 U/mL glucose–6–phosphate dehydrogenase, 15.0 μM hemin, 120.0 μg/mL rat liver cytosol as a source of biliverdin reductase, and a complex buffer composed of 100.0 mM KH_2_PO_4_, 2.0 mM MgCl_2_ × 6H_2_O, phenylmethylsulfonyl fluoride, 10.0 μg/mL trypsin inhibitor, 10.0 μg/mL leupeptin, 2.0 μg/mL aprotinin, and 1.0 mM DTT. Two parallel measurements were done, namely a blind and a NADPH line. During the NADPH line, in order to initiate the reaction, 100 µL reduced β-nicotinamide adenine dinucleotide phosphate (β-NADPH) was added into the mixture and it was incubated for 60 min at 37 °C. After 60 min, we applied ice cooling to stop the reaction. For the blind measurements, we created a mixture in which β-NADPH was replaced with measuring buffer. NADPH and blind solutions were measured at 465 nm spectrophotometrically; then, the blind values were subtracted from the NADPH values. The HO activity was represented as the produced amount of bilirubin (nmol) per hour/mg protein.

### 2.4. Measurement of Cardiac GSH + GSSG Content

The LV samples were homogenized first in buffer A (0.25 M sucrose, 20 mM Tris, 1 mM DTT) for 60 s on ice, and centrifuged at 15,000× *g* for 30 min at 4 °C. After centrifugation, 1 mL supernatant was collected and homogenized with 200 µL buffer B (0.25 M sucrose, 20 mM Tris, 1 mM DTT, 0.1 M CaCl_2_). After a 30-min incubation, another homogenization was followed at 21,000 g for 30 min at 4 °C. The clear cytosolic fraction was used for the enzyme assay. In a 96 wells plate, 40 µL sample or standard, 20 µL 5,5′ dithio–bis–2–nitrobenzoic acid and 140 µL NADPH were added. This compilation was incubated for 5 min at 25 °C, after which 10 µL glutathione reductase was added to initiate the reaction. After a 10-min shaking, the 2–nitro–5 thiobenzoic acid formation was monitored at 405 nm. The GSH levels were expressed as nmol/mg protein.

### 2.5. Determination of Cardiac HO-1, TNF-Alpha and cGMP Concentrations

LV tissues were homogenized in phosphate buffer (PBS) (pH7.4) for 20 s and placed into the centrifuge for 20 min at 2500 rpm, at 4 °C. The supernatants were collected carefully and used for ELISA (GenAsia, Shanghai, China) and protein measurements. According to the manufacturer’s instruction, the LV concentrations were determined at 450 nm. The HO-1 levels were expressed as ng/mg protein, the TNF-α values were defined as pg/mg protein, while the cGMP concentration was given as pmol/mg protein.

### 2.6. Measurement of MPO Activity

The rat LV tissues were homogenized twice for 10 s in a dissolvent containing PBS and 0.5% hexadecyltrimethylammoniumbromide (HETAB). The samples were frozen and melted four times, and then centrifuged at 10,000× *g* for 15 min at 4 °C. In a 96 wells plate, 12 µL standard or sample was mixed with 280 µL o-dianisidine dihydrochloride, and the reaction was initiated with 20 µL hydrogen peroxide. The mixture was shaken for 30 s, after which the activity of MPO was detected at 490 nm spectrophotometrically. The values were expressed as µU/mg protein.

### 2.7. Protein Determination

Using a commercial protein assay kit (Bio-Rad Labs), aliquots (20 μL) of the diluted samples were mixed with 980 μL of distilled water, with 200 μL Bradford reagent added to each sample. After mixing and following a 10 min incubation, the samples were assayed spectrophotometrically at 595 nm. The protein level was expressed as mg protein/mL.

### 2.8. Statistical Analysis

The results are expressed as the means ± S.E.M. The differences between groups were calculated using a one-way ANOVA followed by Tukey post-testing, and *p* ≤ 0.05 was considered as significant.

## 3. Results

### 3.1. Changes in Serum Testosterone

At the end of the experimental period, the serum testosterone levels were measured in both young and aged animals. As expected, the hormone level was significantly reduced by the aging process, and it was undetectable in the castrated (CAS and CAS + *Cypr*) groups. Treatment with testosterone undecanoate resulted in the restoration of the androgen level in young animals, and caused a further enhancement in the case of aged animals. The data are presented in Table 2.

### 3.2. Measurement of Cardiac HO Activity and HO-1 Concentration

**Figure 2:**
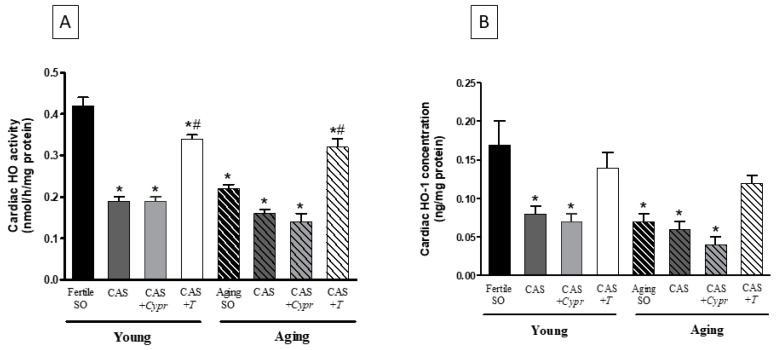
(**A**) The effects of aging, surgical castration, and testosterone replacement therapy on the cardiac HO activity (HO; expressed as nmol bilirubin/h/mg protein). The results are shown as the means ± S.E.M. n = 7–9. (**B**) The effects of aging, surgical castration, and testosterone replacement therapy on the cardiac HO-1 concentration (HO-1; expressed as ng/mg protein). The results are shown as the means ± S.E.M. n = 4–7. * *p* < 0.05: Statistical significance relative to the Young/SO fertile group, ^#^ *p* < 0.05: Statistical significance between age-matched CAS and CAS + *T* groups, CAS = surgical castration, T = testosterone replacement therapy, *Cypr* = cyproterone acetate treatment, and SO = sham operation.

The efficacy of the aging process, surgical castration, and hormone replacement therapy was examined on the antioxidant HO enzyme system. As shown in Figure 2A, a significant decrease of the cardiac HO activity was observed in both aged animals and castrated rats. However, no differences existed between the CAS and CAS + *Cypr* groups. Supplements of testosterone for 6 weeks enhanced the HO activity, which was statistically similar in both young and aging CAS + *T* groups.

In agreement with the HO activity results, the testosterone deficiency in aged and castrated animals resulted in a significant reduction in the HO-1 concentration compared with the young/fertile SO rats. The reduced HO-1 values were compensated by testosterone therapy in both CAS + *T* groups. The data are presented in Figure 2B.

### 3.3. Determination of Cardiac GSH + GSSG Content

**Figure 3:**
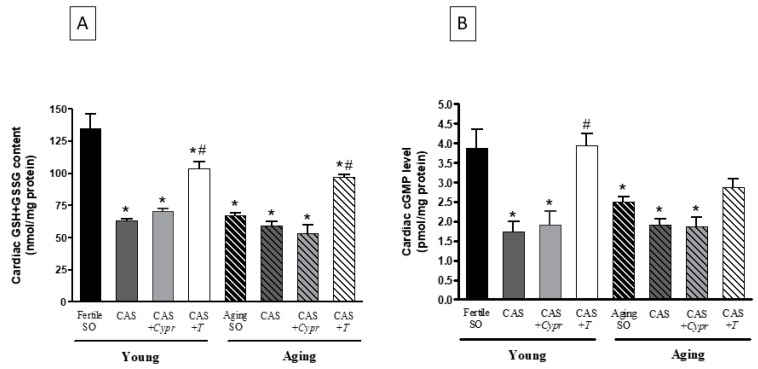
(**A**) The effects of aging, surgical castration, and testosterone replacement therapy on the ratio of the reduced/oxidized glutathione content (GSH + GSSG; expressed as nmol/mg protein). The results are shown as the means ± S.E.M. n = 8–9. (**B**) The effects of aging, surgical castration, and testosterone replacement therapy on the cardiac cyclic guanosine monophosphate level (cGMP; expressed as nmol/mg protein). The results are shown as the means ± S.E.M. n = 8–9. * *p* < 0.05: Statistical significance relative to the Young/SO fertile group, ^#^ *p* < 0.05: Statistical significance between age-matched CAS and CAS + *T* groups, CAS = surgical castration, T = testosterone replacement therapy, *Cypr* = cyproterone acetate treatment, and SO = sham operation.

As shown in Figure 3A, the fertile SO rats exhibited the highest GSH + GSSG value, whereas a significant decrease was found in testosterone-deficient (aging and CAS) animals. As a result of the 6-week-testosterone treatment, a significant improvement in the cardiac antioxidant status was detected in both young and aged rats.

### 3.4. Evaluation of Cardiac cGMP Level

The cardiac cGMP level reached the highest value in the Young/fertile SO rats, while testosterone deprivation resulted in a significant reduction in the CAS and CAS + *Cypr* groups. Similarly, in the aging groups with a lower testosterone level, the cardiac cGMP levels were significantly lower compared to the young/fertile SO rats. Exogenous testosterone replacement therapy was efficient in both young and aged animals. The data are presented in Figure 3B.

### 3.5. Cardiac MPO Activity

**Figure 4:**
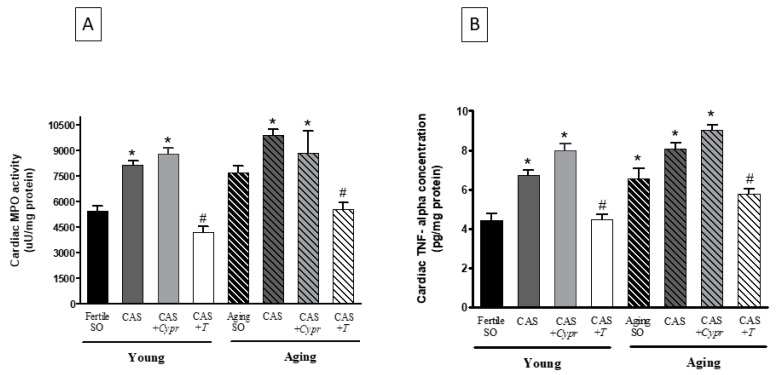
**(A**) The effects of aging, surgical castration, and testosterone replacement therapy on the cardiac myeloperoxidase enzyme activity (MPO; expressed as uU/mg protein). The results are shown as the means ± S.E.M. n = 7–10. (**B**) The effects of aging, surgical castration, and testosterone replacement therapy on the cardiac tumor necrosis factor-alpha concentration (TNF-α; expressed as pg/mg protein). The results are shown as the means ± S.E.M. n = 7–9 * *p* < 0.05: Statistical significance relative to the Young/SO fertile group, # *p* < 0.05: Statistical significance between age-matched CAS and CAS + *T* groups, CAS = surgical castration, T = testosterone replacement therapy, *Cypr* = cyproterone acetate treatment, and SO = sham operation.

Figure 4A presents the MPO values measured at the end of the experimental period. As expected, testosterone deficiency caused by aging or surgical castration resulted in a significant increase in the inflammatory processes, as shown by the elevation of the MPO activity. However, testosterone-treated rats possessed lower values compared to the untreated counterparts.

### 3.6. Cardiac TNF-α Concentration

Similar to the MPO activity values, the TNF-α concentrations were significantly increased with regards to the aged and castrated animals. 6 weeks of testosterone therapy was able to mitigate these elevated values in both the young and aging CAS + *T* groups. The data are presented in Figure 4B.

### 3.7. Serum GOT, GPT, Cholesterol, and Triglyceride Concentrations

A similar result was observed in the case of the GOT and GPT concentrations when the aging status resulted in a significant increase in the GOT and GPT values compared to the young/fertile SO rats.

The serum cholesterol and triglyceride concentrations were enhanced in castrated and aged rats, and the highest values were observed in the case of the CAS + *Cypr* groups. We can summarize that hormone replacement therapy was able to ameliorate the metabolic parameters, except for the cholesterol values of aged rats where testosterone treatment did not mitigate the castration-induced adverse changes.

## 4. Discussion

In the present study, the relationship between the testosterone level, oxidant/antioxidant homeostasis, as well as the inflammatory parameters has been determined. Our results show that testosterone deprivation caused by the aging process or surgical castration deteriorated the antioxidant status of LV and resulted in higher levels of inflammatory parameters via the modulation of the HO system. Hormone replacement therapy was observed to prevent oxidative damage, which was effective in both young and aged rats.

A number of studies support the view that endogenous sex hormones possess an important role in the cardiovascular system and in the maintenance of the normal lipid profile [12]. Similar to estrogen deficiency [13,14], a low testosterone level is an independent cardiovascular risk factor and produces adverse effects on cardiac function due to the presence of androgen receptors in the myocardium [15]. Based on the antioxidative effects of sex hormones, a decline in the testosterone level causes homeostatic shifts toward the oxidative stress [16]. Our current results show that the testosterone level could be aligned with the antioxidant/oxidant state. We found an age-related decrease in the testosterone level of aged animals (~63%), while testosterone concentrations in castrated animals dropped to zero. Hormone replacement therapy restored testosterone levels in young animals, and exceeded the physiological value in the aged group. To provide evidence that testosterone absence contributes to the deterioration of antioxidant defense mechanisms, the role of the cardiac HO enzyme system has been analysed. HO enzymes occur in many mammalian tissues, and possess antioxidant and anti-inflammatory properties via the release of its by-products (biliverdin/bilirubin, CO, and free iron). The relationship of the HO system and the testosterone level in the myocardium has not been previously investigated; however, the effects of estrogen, the other sex hormone, on the HO system has been widely distributed in the cardiovascular system [17,18,19,20]. In our previous study, we verified that estrogen deprivation related to the aging process or to pharmacologically/surgically-induced hormone deficiency resulted in a significant decrease of the cardiac HO activity and expression [13]. The onset of cardiovascular complications coincided with the reduced HO activity and expression, as well as with a linear increase in the MPO enzyme activity and in the concentrations of the TNF-α pro-inflammatory cytokine. Demirbag et al. associated the sex hormones with the total antioxidant capacity and found a strong correlation between the antioxidants, testosterone, and estrogen hormones [21]. Beside sex hormones, the aging process with its multifactorial properties influences the oxidant/antioxidant homeostasis. Oxidative stress and the reduction of antioxidant mechanisms are general in aged animals, promoting the development of cardiovascular pathological processes [22]. In agreement with previous observations, our results clearly show that the aging process contributed to the deterioration of the antioxidant state via the diminishment of the HO system. Surgical castration induced a significant decrease in the cardiac HO enzyme activity and HO-1 concentration in both young and aged animals, while cyproterone acetate-treated rats possessed the lowest HO values as a result of the absolute block of androgen production. Similar to estrogen replacement therapy [23], we observed a protective role of testosterone administration against oxidative damages. Exogenous testosterone-induced amelioration was effective in both young and aged animals. It has been proven that endogenous sex hormones protect the cells form oxidative damage; however, conflicting results have been observed in connection with the potential antioxidant role of exogenous testosterone. Klapcinska et al. found that castration negatively influenced the antioxidant status of the rat heart, which was further decreased due to androgen replacement therapy [24]. In a similar experimental design, Zhang et al. investigated the regulation of the cardiac redox state. They found that testosterone deficiency induced oxidative stress, while exogenous testosterone ameliorated superoxide dismutase and glutathione peroxidase enzyme activities in the cardiomyocytes [7]. Under oxidative stress, various antioxidant defense systems contribute to scavenging ROS; however, the activities of these systems depend mostly on the duration of exposure and the intensity of the oxidative stress. While the defense pathways could be adversely affected, the HO system, by its antioxidant, anti-inflammatory, anti-proliferative and vasodilative properties, clearly proved the beneficial effect of testosterone-mediated cardioprotective processes. Beside animal studies, the testosterone level in humans has been also examined and associated with their cardiovascular health. Hormonal decline increases the risk of myocardial infarction [25], coronary heart disease [26] and promotes further comorbidities [27]. Mancini et al. reported that testosterone replacement restored the antioxidant capacity to normal values, suggesting the protective role of the hormone [28]. In order to verify our hypothesis about the protective role of testosterone replacement therapy, the cardiac GSH/GSSG content has been also measured. Our study indicates that the aging process and testosterone deficiency resulted in a significant decrease in the level of GSH/GSSG as compared to fertile animals; however, exogenous hormone administration improved these reduced values.

In line with the antioxidant capacity, the inflammatory parameters were also changed in various testosterone-saturated conditions. It has been well documented that the incidence and progression of chronic diseases show a strong correlation with the high levels of inflammatory markers [29]. Testosterone deprivation-induced inflammatory processes correlate with the increase of the adverse metabolic parameters. Inflammatory and metabolic markers alone or together promote the incidence and progression of various pathological cardiovascular processes [30]. Haring et al. verified that the plasma level of sex steroids in men shows a negative correlation with inflammatory parameters [31]. Whereas inflammation is elevated in hypogonadal men, testosterone therapy is able to attenuate the pathological values. In accordance with the human results, we have also proved that exogenous testosterone treatment mitigated the MPO enzyme activity and the concentration of TNF-α, which were increased in testosterone-deficient groups. A growing body of evidences have demonstrated that HO-1 and its metabolites play a key role in the attenuation of pro-inflammatory cytokines. HO-1 reduces the production of TNF-α, interleukin (IL)-1β, and IL-6 vice versa, while the level of anti-inflammatory cytokines are elevated due to the protective effects of HO [30]. Kapturczak et al. verified in HO-1 knockout mice that the absence of HO-1 correlated with the inflammatory state [32]. Our findings are consistent with the anti-inflammatory role of HO-1. The cardiac HO activity and HO-1 concentration were reduced in line with elevated TNF-α values in aged or castrated animals, whereas hormone replacement therapy improved the antioxidant/inflammatory balance. We can summarize that a similar association could be observed between the HO and MPO activity and in the interplay of HO and TNF-α. Sexual hormones mediate their effects via binding to their receptors, and they activate DNA-binding or non-DNA-binding-dependent actions [33]. Among steroid hormones, testosterone is a potential inductor of HO enzymes and causes antioxidant, anti-inflammatory, and anti-proliferative effects via releasing CO and bilirubin biological products. It has been well documented that the effects of CO resemble those of NO due to their common signaling pathways. Androgen receptor-bound testosterone triggers the ERK1/2-MAPK cascade to regulate the nitric oxide synthase (NOS)/NO pathway and mediate its effect affected by cGMP [34]. However, the amount of CO has not been measured in this study; our results related to the cGMP content support this interaction. The production of the anti-inflammatory compounds biliverdin/bilirubin and CO play a major role in counteracting inflammatory reactions. Similar to the nitric oxide (NO)-cGMP signalling mechanism, CO-regulated cGMP production has a beneficial role against vascular inflammation and in many cardiovascular complications [35]. Rizzo et al. reported that reduced NO-cGMP signalling is a trigger of vascular inflammation and insulin resistance [36]. Consistent with the data of the HO activity, HO-1 concentration as well as the inflammatory markers, our findings present reduced cGMP values in the lack of testosterone. The protective role of exogenous therapy against inflammation has also been reflected in the elevated level of cGMP. Beside the oxidative and inflammatory effects, metabolic parameters also increase the risk of cardiovascular diseases. Changes in the lipid profile are correlated with the testosterone level. In agreement with Rouver et al. [37], we demonstrated that the concentrations of cholesterol, triglyceride, and GOT levels were increased as a result of testosterone deficiency, whereas exogenous hormone therapy ameliorated the adverse values. Advancing age increased the lipid parameters in themselves, and they were further elevated in the aged-castrated animals. Similar to the antioxidant and anti-inflammatory properties, the metabolic parameters were also improved as a result of testosterone administration.

In summary, we can conclude that the HO system is involved in testosterone-mediated mechanisms in both young and aged animals. In addition to the effects of the endogenous sex hormone, exogenous hormone therapy promotes antioxidants and anti-inflammatory processes via the enhancement of the HO enzymes. Although aging processes are associated with oxidative damages, hormonal treatment restores the adverse antioxidant/inflammatory balance in the heart, which may improve the cardiovascular health of testosterone-deficient patients.

## Figures and Tables

**Table 1:** Number of the animals per group (n). CAS = surgical castration, T = testosterone replacement therapy, *Cypr* = cyproterone acetate treatment, and SO = sham operation.

Parameters	Young				Aging			
	Fertile	CAS	CAS + *Cypr*	CAS + *T*	SO	CAS	CAS + *Cypr*	CAS + *T*
HO activity	8	8	9	8	9	8	7	9
HO-1 concentration	5	6	4	5	5	5	5	5
GSH + GSSG content	9	9	8	9	8	8	8	9
cGMP level	6	5	4	5	8	6	5	9
MPO activity	8	8	8	8	7	8	7	10
TNF-alpha concentration	8	9	8	7	7	8	8	9
Testosterone level	5	6	4	7	7	6	7	8
GOT level	7	6	4	7	8	7	7	7
GPT level	7	4	4	7	8	4	6	8
Cholesterol level	7	6	4	7	8	7	6	7
Triglyceride level	7	5	4	5	8	6	6	6

**Table 2:** Changes in the serum levels of testosterone (expressed as: ng/dL), glutamic oxaloacetic transaminase (GOT: expressed as U/L), glutamic-pyruvic transaminase (GPT: expressed as (U/L), cholesterol (expressed as mmol/L), and triglyceride (expressed as mmol/L). The results are shown as the means ± S.E.M. n = 4–10. * *p* < 0.05: Statistical significance relative to the Young/SO fertile group, ^#^ *p* < 0.05: Statistical significance between age-matched CAS and CAS + *T* groups, CAS = surgical castration, T = testosterone replacement therapy, *Cypr* = cyproterone acetate treatment, and SO = sham operation.

	Young				Aging			
	Fertile	CAS	CSA + *Cypr*	CAS + *T*	SO	CAS	CAS + *Cypr*	CAS + *T*
Testosterone (ng/dl)	237.40 ± 36.58	0 ± 0 *	0 ± 0 *	149.86 ± 21.19 ^#^	95.90 ± 12.26 *	0 ± 0 *	0 ± 0 *	364.50 ± 50.19 *^,#^
GOT (U/l)	94.57 ± 3.02	106.67 ± 11.85	142.50 ± 23.50	105.14 ± 3.91	184.75 ± 18.72 *	163.86 ± 6.11 *	163.00 ± 10.64 *	145.29 ± 13.03
GPT (U/l)	56.00 ± 1.60	5.00 ± 1.22	77.00 ± 6.23	59.57 ± 1.85	94.00 ± 10.10 *	88.25 ± 3.66 *	90.67 ± 6.38 *	79.13 ± 5.11
Cholesterol (mmol/l)	2.20 ± 0.13	2.78 ± 0.13	3.09 ± 0.12	2.29 ± 0.08	3.86 ± 0.44	4.65 ± 0.63 *	4.81 ± 0.86 *	4.46 ± 0.51 *
Triglyceride (mmol/l)	0.92 ± 0.13	1.01 ± 0.06	2.84 ± 0.39 *	1.28 ± 0.13	1.62 ± 0.31	1.69 ± 0.27	1.75 ± 0.30	1.33 ± 0.15
